# Periodontitis, age-related diseases and diabetes in an endocrinological outpatient setting (PARADIES): a cross-sectional analysis on predictive factors for periodontitis in a German outpatient facility

**DOI:** 10.1007/s00592-021-01838-z

**Published:** 2022-01-04

**Authors:** Stefan Kabisch, Oda Sophia Hedemann, Andreas F. H. Pfeiffer

**Affiliations:** 1 Department of Endocrinology, Diabetes and Nutrition, Campus Benjamin Franklin, Charité University Medicine, Hindenburgdamm 30, 12203 Berlin, Germany; 2 Deutsches Zentrum Für Diabetesforschung E.V., Geschäftsstelle Am Helmholtz-Zentrum München, Ingolstädter Landstraße 1, Neuherberg, 85764 Neuherberg, Germany; 3 Praxis Am Posthof, Hameln, Germany

**Keywords:** Periodontal disease, Periodontitis, Risk factor, Prediction, Diabetes mellitus

## Abstract

**Background:**

Diabetes mellitus (DM) is a risk factor for periodontitis. Data on risk-modifying factors for periodontitis in diabetes patients are limited.

**Aims:**

We tested whether sex, age, type of diabetes, metabolic state, comorbidities, complications, measures of well-being and quality of life are predicting periodontitis in a German diabetes outpatient cohort.

**Methods:**

In total, 1180 out of 1293 participating DM patients completed questionnaires on quality of life, dental hygiene and health. All patients also filled out a modified version of the periodontitis risk questionnaire by the American Association for Periodontology, from which the status of “assumed periodontitis” was deducted. In a subset of participants (*n* = 461), we measured or inquired the most recent Community Parodontal Index (CPI), providing an objective measure for clinically diagnosed periodontitis. For all subjects, DM history and phenotype, major metabolic parameters (HbA1c, BMI, LDL and total cholesterol levels), general health risk factors, comorbidities and medication were collected.

**Results:**

Clinically diagnosed (CPI > 2) and assumed periodontitis was detected in 60–67% of our patients. Male sex and oral health-related quality of life were associated with clinically diagnosed periodontitis. Male sex, age, smoking, dental hygiene, dental control and diabetes-related quality of life independently predicted assumed periodontitis.

**Conclusion:**

In DM patients, quality of life and lifestyle factors which systemically alter microvascular and immunological functions seem to predict periodontitis. Further studies are needed for replication and for pathomechanistic clarification.

**Supplementary Information:**

The online version contains supplementary material available at 10.1007/s00592-021-01838-z.

## Introduction

Diabetes mellitus (DM) is a pandemic metabolic disorder, which slowly, but continuously contributes to premature death and various kinds of comorbidities. Cardiovascular disease, cancer and other long-term complications are the typical fate of DM patients. Periodontitis, the sixth main complication of DM, is a chronic inflammatory destruction of the connective tissue apparatus, which dissolves the periodontium and leads to resorption of the alveolar bone. DM is also associated with chronic inflammation, partially explained by visceral fat accumulation, and a slight impairment of immune processes [1]. Periodontitis requires a localized bacterial infection, and severe progression is connected to smoking, stress and DM [2–4]. Patients with DM have a higher risk for periodontitis compared to persons with normal glucose metabolism [5]. DM duration is positively associated with this risk [6, 7]. Also, poor DM control is associated with higher risk for periodontitis [8], but there is only limited data for DM patients with sufficient treatment [9]. The pathomechanistic connection between DM and periodontitis is believed to be bidirectional [7, 10, 11]. However, there is no consensus, in which factors within DM specifically contribute to periodontitis and factors of host susceptibility are not fully understood [12].

Studies did not show an alteration of the parodontal microbiome,^1^ but the DM-associated elevated accumulation of advanced glycation endproducts (AGE), impairments of microcirculation and increased appearance of inflammatory mediators may explain the disposition for periodontitis [7, 13, 14]. This pattern of DM sequelae is a common hypotheses on how periodontal decay is facilitated in an hyperglycemic environment [15]. Additional factors may be of secondary nature to DM onset, e.g., xerostomia as a side effect of typical antihypertensive medication [9].

The observations on the interaction of immune reactions and glycemic state are assumed to be primarily independent of the type of DM, as an inflammatory cascade in response to parodontal bacteria was seen in patients with T1DM as well [16].

On the other hand, periodontitis as a primary condition can worsen insulin resistance, which is more common in T2DM [17]. Parodontal treatment was reported to improve the HbA1c level by an average of 0.46% points [18].

Worldwide, severe periodontitis affects at least about 5–15% of the entire population [19, 20] with rapidly increasing prevalence [21, 22]. These estimations are questionable, as there is no consistent examination protocol for this very common disease [23], and different index systems are used [24]. The German Oral Health Study (Deutsche Mundgesundheits-Studie V = DMS-V) described frequency, age distribution and co-factors of periodontitis in a representative German population sample. The most recent—fifth—follow-up assessment covers the observational period between 1989 and 2014 [25]. As the DMS-V has only limited data for the highly affected group of DM patients, our PARADIES study aims to assess predictors and risk indicators for periodontitis in a community-derived sample of DM patients. We want to address the (independent) role of sex, age, age of DM onset, DM duration and type of DM, typical DM complications, metabolic control, oral hygiene, smoking, regular dentist checkups, and measures of general and oral-specific quality of life.

## Material and methods

We recruited among the clinical practice “Praxis am Posthof” in Hameln and asked all adult patients (age > 18 years) with known DM, visiting the facility between July 1, 2017, and September 30, 2017, for their participation in the cross-sectional study. Every patient (age > 18 years) which came to the quarter checkup was asked to participate in the study. Presence of diabetes was defined based on diabetes medication or evident plasma glucose levels above 126 mg/dl in a previous fasting sample. All participants provided informed consent, the study protocol was approved by the ethics commission of the Charité in Berlin prior to recruitment (April 25, 2017, EA4/040/17).

All consenting participants were asked to provide a set of questionnaires. The “Problem Areas In Diabetes” (PAID) [26], which we used in its short German version [27], covers aspects of quality of life, which are connected to diabetes symptoms, health care and treatments. The questionnaire on the Oral Health Impact Profile (OHIP-G14) [28], which we also used in its shortened and translated version (MLQ) [29, 30], was chosen as a tool to assess the individual pattern of dental care, dental status and dental symptoms. The self-report test for periodontitis risk (American Association of Peridontology; AAP) [31], which was provided in the revised German version [32], specifically inquires information about the currently known risk factors for periodontitis. It can be used to estimate the presence and severity of periodontitis in individual patients. A customized questionnaire for self-reporting oral hygiene was added in order to cover more aspects of dental care than the previous tools.

Participants granted access to their medical file of the “Praxis am Posthof,” providing data on medical history (diagnoses, blood parameters, anthropometry) including, but not limited to DM. Between August 10 and 24, 2017, a subset of participants was invited to undergo a physical examination in the “Praxis am Posthof.” Selection of this subset of patients was done in a randomized fashion applied to each 20-min time frame, for which diabetes patients of our clinic were scheduled. Thus, a continuous series of examinations could be conducted. The examination including assessment of the DMFT (“decayed, missing or filled teeth”) index [33] and the Community Periodontal Index (CPI) [34]. At the time of the conceptualization of PARADIES, there was no accepted diagnostic algorithm for periodontitis. We therefore decided to use the CPI, as it is an easy and quick assessment, which could be done in an outpatient non-dentist setting. It is also a common index in the everyday routine in German dental offices, allowing comparisons with medical information provided by our patients’ dentists (see below).

CPI examination was done on a regular medical examination couch using magnifying glasses (magnification 2.7) and lighting for visual inspection. Dental mirrors, probes, tweezers and WHO probes were used once only. Saliva was not sucked off during examination; patients were asked to swallow in between. We did not perform X-ray imaging and did not use existing X-ray images. Number of teeth was assessed including wisdom teeth. Numbers of dental crowns and fillings were not counted. This examination was done by an experienced dentist (OH).

All subjects were also asked to provide medical information about their oral health status from the most recent visit at their personal dentist, including the CPI.

For the CPI-based analysis in this paper, patients were classified as clinically diagnosed periodontitis positive with a CPI > 2 in at least one oral sextant. In accordance with the original AAP risk score, which classifies patients as “at elevated risk,” when surpassing 21 points, and “at high chance for periodontitis,” when surpassing 26 points, we categorized all patients with a score above 21 points as “assumed periodontitis.” In our cohort, 59.8% (276/461) subjects had a CPI > 2, and 66.6% (786/1180) had a risk score > 21 points.

### Statistical analyses

Due to the exploratory nature of this study, a sample size calculation was not done. We did not desire to replicate a certain frequency of periodontitis cases or diabetes complication, as the study setting in an endocrinological outpatient clinic is completely novel for the investigation of periodontitis and we aimed to cover all eligible, available and willing patients within three months of clinical practice.

We examined sex, type of diabetes, diabetes onset and duration, DM-related comorbidities (hypertension, depression), microvascular complications (neuropathy, nephropathy, retinopathy), HbA1c, cholesterol levels, body weight, diabetes medication, dental hygiene, smoking as well as diabetes-related and oral health-related quality of life as predictors of clinically diagnosed and assumed periodontitis. We also analyzed the association between smoking and HbA1c with severity of clinically diagnosed and assumed periodontitis. Given the frequent absence of normal distribution in the Kolmogorov–Smirnov test, we decided to conduct nonparametric tests (Mann–Whitney tests) for group-wise comparisons. For correlations, the nonparametric Spearman approach was chosen. Finally, multivariate logistic regression analysis was used in order to determine an adjusted contribution of several factors to the risk for periodontitis. As supplementary analysis, the modified AAP risk score was tested for its suitability as surrogate parameter for clinically diagnosed periodontitis (CPI) by receiver operating characteristic (ROC) analysis.

All data are presented as medians with interquartile ranges (IQR). The results were considered significantly different if *p* < 0.05. All statistical analyses were performed using SPSS for Windows program version 25.0 (SPSS Inc, Chicago, IL, USA).

## Results

### Cohort structure

The PARADIES study included 1293 patients with DM, aged 18–93 years, who consented to the participation. In total, 375 patients with DM declined their consent or were ineligible. Participants and non-participants did not differ by sex distribution or age, but T1DM patients more likely took part in the trial than declining participation.

Out of the 1293 participating patients, 84.2% (*n* = 1089) had T2DM and 14.8% (*n* = 191) had T1DM. 1% (*n* = 13) had type 3 diabetes and were discarded from all following analyses, resulting in a sample of 1280 subjects. Mean age was 67 ± 12 years for T2DM patients and 48 ± 16 years for T1DM patients. Sex distribution was similar for T1DM and T2DM cases (45.0% (*n* = 86/191) and 45.5% (*n* = 495/1089) female, respectively).

In total, 895 subjects allowed access to their dentist’s medical records; out of these, 639 subjects actually provided information on their oral health from their personal dentist. In total, 370 dentist reports included a CPI measurement. In total, 150 randomly selected subjects underwent the physical examination by a dentist in our study facility (OH), which included assessing DMFT index and CPI. Thus, 461 subjects having at least one tooth were either examined directly for or provided CPI data from their dentist. Fifty-seven subjects provided DMFT, CPI and number of teeth by their original dentist and the dentist in our study facility. These assessments were consistent. Comparison of subjects with and without directly measured CPI data did not show significant differences in age, sex, BMI or type of DM, while subjects with reported CPI (from the original dentist) were significantly younger (by 2.5 years) and more likely to be T1DM patients (23% vs. 15%) compared to those without a dentist report.

However, patients who did not allow access to their dentist’s records (*n* = 398) had significantly higher PA risk scores, poorer dental hygiene, more infrequent dentist visits compared to those with a dentist report, but there were no differences in age, sex, smoking frequency or metabolic parameters (data not shown).

**Fig. 1:**
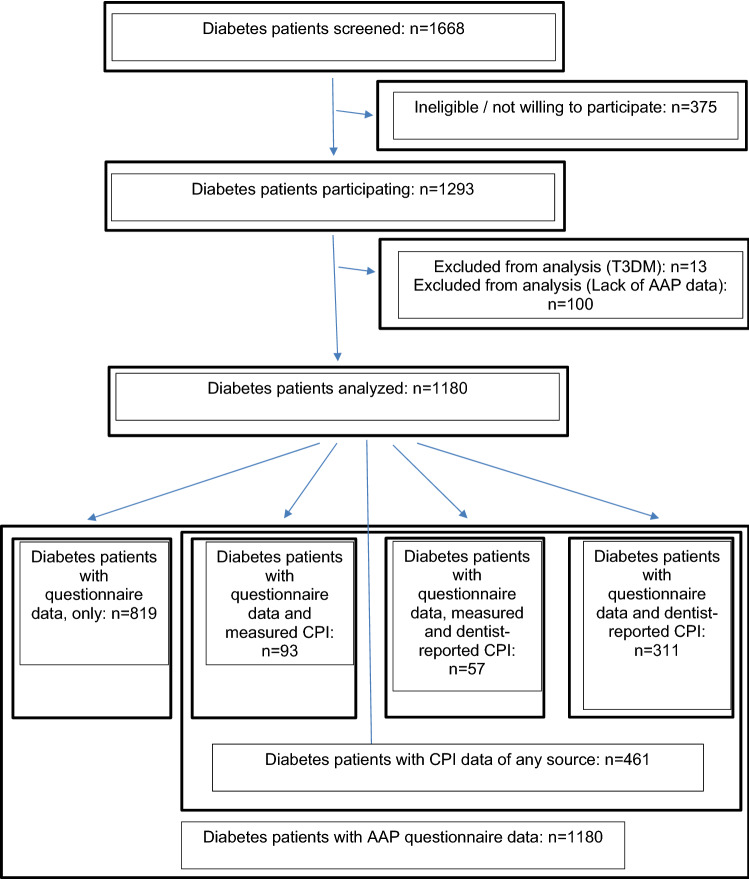
Cohort structure

Fifteen out of 150 examined patients, 42 of 610 patients with dentist’s data and 137 out of 1141 patients with questionnaire data on oral health (10/178 with T1DM; 127/963 with T2DM, *X*^2^ test: *p* < 0.001) were edentulous. The cohort structure is shown in Fig. 1.

### Modulating factors of periodontitis risk

- Sex, type of DM and DM duration
  Frequencies of both clinically diagnosed periodontitis and assumed periodontitis did not differ significantly between the T1DM and T2DM group. In both T1DM and T2DM, men were more likely affected by assumed periodontitis; in T2DM, also clinically diagnosed periodontitis was significantly more common among men than women (Table 1). In both T1DM and T2DM, men had a significantly higher PA risk score than women (23 ± 3 vs. 22 ± 3; *p* = 0.016; and 23 ± 2 vs. 22 ± 2; *p* < 0.001). This minor difference is plausibly explained by the questionnaire scheme, granting men an extra point.
  In T1DM patients, those with assumed periodontitis were significantly older and had a higher age at DM onset compared to assumed-to-be-negative patients; for clinically diagnosed periodontitis, a similar difference in age of onset was detected. In T2DM patients, assumed periodontitis cases were significantly older than controls. Comparison of T2DM patients based on clinical periodontitis diagnosis did not show significant differences (Table 2).
  The AAP risk score attributes 0 to 10 points depending on age. Individual risk scores correlated with age and age of onset in T1DM (linear; *r* = 0.319; *p* < 0.001 and *r* = 0.301; *p* < 0.001), but not T2DM. There was no correlation with DM duration.
- Association with DM complications and comorbidities
  In T1DM only, assumed periodontitis was associated with hypertension, while other typical DM complications were distributed similarly among periodontitis patients and their healthy counterparts. In T2DM, patients with assumed periodontitis were significantly more likely affected by neuropathy and nephropathy compared to subjects with an AAP risk score below 22 points. Depression or retinopathy was distributed equally among periodontitis cases and controls (Table 3). In both T1DM and T2DM, the number of diabetic complications (neuropathy, nephropathy, retinopathy) increased the risk for clinically diagnosed or assumed periodontitis (data not shown).
- Interaction of metabolic control and periodontitis risk
  In both T1DM and T2DM patients, cases and non-cases did not differ by HbA1c or BMI. T2DM patients with assumed periodontitis (vs. non-affected T2DM patients) were treated with significantly more types of medication and more often with insulin, even though the difference is numerically small. Assumed periodontitis in T2DM patients was also associated with significantly, but just slightly lower levels of total and LDL cholesterol compared to non-affected T2DM patients (Table 4). Stratification by HbA1c median split did not reveal a significant impact of this parameter on periodontitis frequency (Supplemental Table 1).
- Role of oral hygiene, smoking and dentist appointments for periodontitis risk
  Sufficient oral hygiene was defined by at least twice daily teeth brushing and use of at least tooth brush, tooth paste and another hygiene tool. Also, smoking is considered a typical risk factor for periodontitis and thus an item of the AAP risk score. At least annual dentist consultations were considered as sufficiently frequent. In both T1DM and T2DM, patients with assumed but not clinically diagnosed periodontitis were significantly more likely to have poor dental hygiene and to be smokers. Clinically diagnosed periodontitis in T1DM and assumed periodontitis were significantly less likely in T1DM and T2DM patients with at least annual dentist appointments compared to their counterparts (Table 5). When using the AAP risk score (but not CPI) as periodontitis assessment, smoking was significantly associated with severity of periodontitis in T1DM and T2DM patients (Supplemental Table 2).
- General and oral-specific quality of life as determinant of periodontitis risk
  T1DM and T2DM participants with assumed but not clinically diagnosed periodontitis had significantly higher MLQ scores than unaffected patients. T1DM subjects with assumed periodontitis had also higher PAID scores than unaffected patients (Table 6).
  Based on ROC analyses, in T1DM (but not T2DM) subjects, PAID-5 and MLQ, predicted assumed periodontitis defined by the modified AAP risk score (AUROC of 0.627 and 0.652, respectively), but not defined by CPI. There were also significant weak correlations between MLQ score and AAP risk score in T1DM (*r* = 0.335; *p* < 0.001) and T2DM (*r* = 0.173; *p* < 0.001), but not for the PAID score (data not shown).
- Logistic regression analysis
  In the adjusted logistic regression analysis, integrating all previous predictor candidates, clinically diagnosed periodontitis was only determined by sex and MLQ with borderline significant contribution of DM duration. Significant predictors for assumed periodontitis were male sex, age and smoking—which are part of the AAP questionnaire, as well as poor oral hygiene, infrequent dentist appointments and a higher PAID score (Table 7).

**Table 1:** Frequency of clinically diagnosed and assumed periodontitis among diabetes types

Diabetes type	T1DM		T2DM	
Clinically diagnosed periodontitis (CPI; n (%))	48/80 (60.0%)		228/381 (59.8%)	
Clinically diagnosed periodontitis (CPI; n (%)), by sex	♂: 26/39 (66.7%)	♀: 22/41 (53.7%)	♂: 129/199 (64.8%) *	♀: 99/182 (54.4%) *
Assumed periodontitis (AAP risk score; n (%))	106/169 (62.7%)		680/1011 (67.3%)	
Assumed periodontitis (AAP risk score; n (%)), by sex	♂: 70/93 (75.3%) ***	♀: 36/76 (47.4%) ***	♂: 413/554 (74.5%) ***	♀: 267/457 (58.4%) ***

*AAP* risk score by the American Association of Periodontology; *CPI* Community Parodontal Index; clinically diagnosed periodontitis: CPI > 2; assumed periodontitis: modified AAP risk score > 21 points; two-sided *X*^2^ tests comparing sexes are significant with * *p* < 0.05; ** *p* < 0.01; *** *p* < 0.001

**Table 2:** Age, diabetes duration and age at diabetes onset of clinically diagnosed and assumed periodontitis cases in T1DM and T2DM patients

	T1DM with periodontitis	T1DM without periodontitis	T2DM with periodontitis	T2DM without periodontitis
Patients with CPI (*n* = 461)				
Age	49 [41;57]	50 [29;58]	68 [57;76]	67 [57;75]
Diabetes duration	19 [9;28]	25 [17;33]	11 [5;17]	11 [6;17]
Age at diabetes onset	30 [21;42] *	23 [11;35] *	54 [46;64]	54 [45;61]
Patients with AAP risk score (*n* = 1180)				
Age	52 [42;61] ***	40 [26;55] ***	69 [59;77] *	66 [57;75] *
Diabetes duration	18 [11;28]	17 [9;33]	12 [6;17]	11 [5;17]
Age at diabetes onset	30 [23;40] ***	17 [10;32] ***	55 [46;63]	54 [46;61]

*AAP* risk score by the American Association of Periodontology; *CPI* Community Parodontal Index; clinically diagnosed periodontitis: CPI > 2; assumed periodontitis: modified AAP risk score > 21 points; median and IQR are given; Mann–Whitney U tests comparing periodontitis vs. non-periodontitis cases; significant differences are indicated by * *p* < 0.05; ** *p* < 0.01; *** *p* < 0.001

**Table 3:** Association between periodontitis and diabetic long-term complications

	T1DM with periodontitis	T1DM without periodontitis	T2DM with periodontitis	T2DM without periodontitis
Patients with CPI (*n* = 461)				
Neuropathy (% cases)	11/48 (22.9%)	8/32 (25.0%)	60/228 (26.3%)	34/153 (22.2%)
Nephropathy (% cases)	5/48 (10.4%)	1/32 (3.1%)	37/228 (16.2%)	16/153 (10.5%)
Retinopathy (% cases)	15/48 (31.3%)	5/32 (15.6%)	18/228 (7.9%)	14/153 (9.2%)
Depression (% cases)	1/48 (2.1%)	2/32 (6.3%)	25/228 (11.0%)	11/153 (7.2%)
Hypertension (% cases)	15/48 (31.3%)	11/32 (34.4%)	178/228 (78.1%)	111/153 (72.5%)
Patients with AAP risk score (*n* = 1180)				
Neuropathy (% cases)	24/106 (22.6%)	11/63 (17.5%)	234/680 (34.4%) ***	73/331 (22.1%) ***
Nephropathy (% cases)	9/106 (8.5%)	4/63 (6.3%)	123/680 (18.1%) **	37/331 (11.2%) **
Retinopathy (% cases)	25/106 (23.6%)	14/63 (22.2%)	74/680 (10.9%)	26/331 (7.9%)
Depression (% cases)	6/106 (5.7%)	3/63 (4.8%)	67/680 (9.9%)	25/331 (7.6%)
Hypertension (% cases)	45/106 (42.5%) *	17/63 (27.0%) *	514/680 (75.6%)	244/331 (73.7%)

*AAP* risk score by the American Association of Periodontology; *CPI* Community Parodontal Index; clinically diagnosed periodontitis: CPI > 2; assumed periodontitis: modified AAP risk score > 21 points; two-sided *X*^2^ tests comparing periodontitis vs. non-periodontitis cases are significant with * *p* < 0.05; ** *p* < 0.01; *** *p* < 0.001

**Table 4:** Association between periodontitis and metabolic state

	T1DM with periodontitis	T1DM without periodontitis	T2DM with periodontitis	T2DM without periodontitis
Patients with CPI (*n* = 461)				
HbA1c (%)	7.4 [6.9;8.2]	7.7 [7.0;8.4]	6.9 [6.3;7.6]	6.9 [6.3;7.8]
BMI (kg/m^2^)	26.7 [23.3;30.5]	26.1 [24.0;28.0]	31.2 [27.1;35.7]	30.6 [27.7;35.4]
Diabetes medication (n)	2 [2;2]	2 [2;2]	2 [1;2]	2 [1;2]
Insulin treatment (n; %)	48/48 (100%)	32/32 (100%)	97/228 (42.5%)	63/153 (41.2%)
Total cholesterol (mg/dl)	205 [174;231]	191 [172;210]	191 [170;223]	196 [166;227]
LDL cholesterol (mg/dl)	121 [92;139]	108 [96;130]	120 [95;146]	125 [100;149]
Patients with AAP risk score (*n* = 1180)				
HbA1c (%)	7.6 [7.0;8.5]	7.5 [6.9;8.4]	7.1 [6.4;7.9]	7.0 [6.3;7.8]
BMI (kg/m^2^)	27.0 [24.0;30.2]	25.3 [23.8;30.2]	30.5 [27.5;34.8]	31.0 [27.6;35.7]
Diabetes medication (n)	2 [2;2]	2 [2;2]	2 [0;2]**	2 [0;2]**
Insulin treatment (n; %)	105/106 (99.1%)	62/63 (98.4%)	359/680 (52.8%) **	148/331 (44.7%) **
Total cholesterol (mg/dl)	200 [176;225]	196 [171;220]	189 [163;223] **	200 [173;231] **
LDL cholesterol (mg/dl)	121 [97;139]	110 [99;133]	116 [94;144] **	123 [96;149] **

*AAP* risk score by the American Association of Periodontology; *CPI* Community Parodontal Index; clinically diagnosed periodontitis: CPI > 2; assumed periodontitis: modified AAP risk score > 21 points; Median and IQR are given. Mann–Whitney U tests comparing periodontitis vs. non-periodontitis cases are significant with * *p* < 0.05; ** *p* < 0.01

**Table 5:** Impact of smoking, oral hygiene and professional dental care on periodontitis diagnosis

	T1DM with periodontitis	T1DM without periodontitis	T2DM with periodontitis	T2DM without periodontitis
Patients with CPI (*n* = 449–461)				
Smoking (%n)	11/45 (22.2%)	7/31 (25.8%)	36/224 (16.1%)	19/149 (12.8%)
Poor oral hygiene (%n)	15/48 (31.3%)	5/32 (15.6%)	78/228 (34.2%)	51/153 (33.3%)
Infrequent dental checks (%n)	9/48 (18.8%)	4/32 (12.5%)	40/228 (17.5%)	21/153 (13.7%)
Patients with AAP risk score (*n* = 1180)				
Smoking (%n)	41/106 (38.7%) ***	8/63 (12.7%) ***	154/680 (22.6%) ***	12/331 (3.6%) ***
Poor oral hygiene (%n)	54/106 (50.9%) ***	6/63 (9.5%) ***	398/680 (58.5%) ***	29/331 (8.8%) ***
Infrequent dental checks (%n)	33/106 (31.1%) *	8/63 (12.7%) *	227/680 (33.4%) ***	61/331 (18.4%) ***

*AAP* risk score by the American Association of Periodontology; *CPI* Community Parodontal Index; clinically diagnosed periodontitis: CPI > 2; assumed periodontitis: modified AAP risk score > 21 points; two-sided X^2^ tests comparing periodontitis vs. non-periodontitis cases are significant with * *p* < 0.05; ** *p* < 0.01; *** *p* < 0.001

**Table 6:** Analysis of parameters for general and diabetes-specific quality of life

	T1DM with periodontitis	T1DM without periodontitis	T2DM with periodontitis	T2DM without periodontitis
Patients with CPI (*n* = 461)				
PAID	6 [2;9]	6 [2;8]	4 [2;8]	4 [1;7]
MLQ	3 [1;10]	7 [1, 15]	7 [2;22]	5 [1;16]
Patients with AAP risk score (*n* = 1180)				
PAID	6 [1;10] **	4 [2;6] **	4 [1;7]	4 [1;8]
MLQ	5 [3;14] **	2 [0;8] **	6 [1;19] ***	3 [0;12] ***

*AAP* risk score by the American Association of Periodontology; *CPI* Community Parodontal Index; clinically diagnosed periodontitis: CPI > 2; assumed periodontitis: modified AAP risk score > 21 points; MLQ: Oral health-related quality of life (Mund-gesundheitsbezoge-ne Lebensqualität); *PAID* Problem areas in diabetes; median and IQR are given. Mann–Whitney U tests comparing periodontitis vs. non-periodontitis cases are significant with * *p* < 0.05; ** *p* < 0.01; *** *p* < 0,001

**Table 7:** Prediction of clinically diagnosed or assumed periodontitis by an adjusted logistic multifactorial model

	Clinically diagnosed periodontitis (CPI)		Assumed periodontitis (AAP risk score)	
Variable	Likelihood ratio *χ* ^2^	*p* value	Likelihood ratio *χ* ^2^	*p* value
Sex	8.475	0.004 **	20.169	< 0.001 ***
Type of diabetes	1.646	0.200	0.318	0.573
Age	1.130	0.288	13.365	< 0.001 ***
Diabetes duration	3.761	0.052	0.305	0.581
Age of onset	Dropped		Dropped	
Neuropathy	0.500	0.480	2.948	0.086
Nephropathy	1.751	0.186	0.924	0.337
Retinopathy	1.254	0.263	0.974	0.324
Depression	1.838	0.175	0.375	0.540
Hypertension	0.540	0.462	1.323	0.250
HbA1c	0.086	0.770	0.468	0.494
BMI	0.354	0.552	0.776	0.378
Total cholesterol	0.209	0.648	0.256	0.613
LDL cholesterol	0.000	0.983	0.061	0.804
Diabetes medication	0.260	0.610	0.490	0.484
Insulin treatment	0.232	0.630	0.573	0.449
Smoking	0.116	0.733	70.591	< 0.001 ***
Poor dental hygiene	0.280	0.597	146.724	< 0.001 ***
Infrequent dentist appointments	0.057	0.811	6.732	0.009 **
PAID	0.405	0.524	4.062	0.044 *
MLQ	4.260	0.039 *	1.494	0.222

*AAP* risk score by the American Association of Periodontology; *CPI* Community Parodontal Index; clinically diagnosed periodontitis: CPI > 2; assumed periodontitis: modified AAP risk score > 21 points; given are Wald’s likelihood ratios in a multivariable logistic regression model for the prediction of clinically diagnosed or assumed periodontitis, * *p* < 0.05; ** *p* < 0.01; *** *p* < 0.001

## Discussion

The PARADIES study demonstrates that patients with DM carry a high risk for suspected or clinically diagnosed periodontitis with a prevalence of 60–67%, replicating results of a previous meta-analysis on cohort studies in T2DM patients [35]. In our assessment, major factors for clinically diagnosed, objectively measured periodontitis seem to be male sex (significantly in T2DM), age of DM onset (in T1DM, only) and oral health-related quality of life (MLQ; in the fully adjusted logistic model). Assumed periodontitis, assessed by using the modified AAP risk score, is connected to male sex, age and smoking—as parameters of the risk score itself—as well as poor oral hygiene and insufficient stomatological monitoring in both T1DM and T2DM, and impaired DM-related quality of life (PAID). Further associations were found with age at DM onset and hypertension (both in T1DM, only), nephropathy, neuropathy and DM medication (all in T2DM, only), as well as quality of life (PAID in T1DM; MLQ in both DM types). In the adjusted model, sex, age, smoking, dental hygiene and control, and DM-related quality of life remained significant, leaving indicators of metabolic state and diabetic long-term complications without statistical significance. Dental care and quality of life are predictive parameters, which are not part of the AAP risk score. However, the modified AAP risk score seems to overestimate the role of smoking and dental hygiene, as only sex, oral health-related quality of life and (trend-wise) DM duration indicated a higher risk of clinically diagnosed periodontitis.

At the time of the conceptualization of the PARADIES study, there was no accepted diagnostic algorithm for the diagnosis of periodontitis [36]. Therefore, we assessed the outcome by clinical assessment of the CPI during the study (*n* = 150), collection of clinical dental data from the patients’ dentists (*n* = 639 reports with *n* = 370 CPI reports) and additional evaluation of periodontitis risk based on a modified version of the AAP questionnaire. The latter was validated against clinically diagnosed periodontitis cases (CPI > 2) in our sample, providing a very moderate approximation of the objective diagnosis. We confirmed by ROC analysis that a risk score of > 21 points is the best cutoff to determine assumed periodontitis (AUROC 0.610; sensitivity 71%, specificity 48%, *p* < 0.001 (see supplemental Fig. 1)).

We also compared CPI measurements, which were done in the Praxis am Posthof, with those done by the patients’ dentists and found an excellent consistency between these examinations.

Our cohort is structurally well comparable to the DMS-V. The DMS-V included patients between 35–44 and 65–100 years of age, while PARADIES subjects were above 18 years of age without exclusions. Subjects of the diabetes cohort PARADIES had a comparable (35–44; 65–74) or slightly higher tooth count than healthy DMS-V subjects in the same age range. Cohorts were comparable with respect to DMFT and oral hygiene. In the age range 65–74, only, PARADIES subjects had poorer oral hygiene and reported more frequent symptom-related dentist visits [25].

Our data only partially support previous studies on risk factors for periodontitis. Recent reports showed that periodontitis is linked to a phenotype pattern of male sex, age, metabolic syndrome and smoking, among which age and smoking seem to be the strongest predictors [37]. As previous studies found that only severity, but not prevalence of periodontitis is linked to T2DM, we also wanted to assess whether the type of DM determines periodontitis risk in our diabetes cohort [38].

Recent data indicate that periodontitis usually occurs after the age of 40 [39]. Other researchers conclude that age itself is not a risk factor, but age-related disorders may facilitate the microbial–inflammatory dysregulation [40]. In our analysis, age was associated with assumed periodontitis, but not with a clinically diagnosis, in both single factor and adjusted logistic regression analysis.

Typical age-related disorders—apart from T2DM, but also in connection with it—are hypertension and depression. These conditions were shown to be more common among periodontitis patients [41, 42], but the impact of hypertension is still questioned [43]. In our study, depression was not a predictor. Hypertension was associated with assumed periodontitis among T1DM subjects in the unadjusted comparison. Thus, macrovascular alterations seem to be of minor relevance for periodontitis onset.

Previous studies indicate that smoking supports onset of periodontitis [2]. We confirmed this finding for assumed, but not CPI-defined periodontitis in both single factor and adjusted logistic regression analysis. Smoking was also associated with periodontitis severity based on the AAP questionnaire, but not when evaluating CPI. Possibly, smoking elicits various orodental and systemic disturbances, lacking specificity for the distinct nature of periodontitis.

In earlier studies, periodontitis was linked to obesity [44] or poor metabolic control [8, 45]. We noticed a more intensified antidiabetic, in particular insulin-based treatment among periodontitis T2DM patients (compared to non-affected T2DM patients), no differences for HbA1c or BMI, and even surprisingly lower cholesterol levels. Insulin treatment is more common among patients with long-term T2DM, but also the typical medication pragmatically used in patients with a certain T2DM subtype. Severe insulin-deficient diabetes (SIDD), as defined by Ahlqvist et al. [46], is a distinct subphenotype, characterized by early beta-cell failure, but also high risk of retinopathy and neuropathy. This would mirror a concomitantly elevated risk of periodontitis. However, our adjusted logistic model did not indicate a relevant risk contribution of metabolic state or medication. Also, HbA1c did not affect periodontitis severity in our sample. Maybe, our exclusive DM cohort contains too few subjects with severely insufficient treatment [9]. Another explanation might be that neither obesity, hyperglycemia, nor dyslipidemia, but rather the less frequent, highly variable microvascular sequelae are more relevant indicators for periodontitis progression. The unexpectedly lower levels of total and LDL cholesterol among periodontitis cases can be attributed to a higher frequency of statin treatment in this group.

Late DM complications (nephropathy, neuropathy, retinopathy) are often connected to impaired microcirculation, which is a pathomechanistic factor for periodontitis. Even though previous studies have found a linkage between loss of teeth or periodontitis and microvascular diabetic complications of the retina [47], the kidneys [48] and the nervous system [49], but also peripheral arterial disease [50, 51], our data only partially replicate these findings. In T2DM patients of our cohort, nephropathy and neuropathy seem to be strongly associated with assumed periodontitis and trend-wise more common in clinically diagnosed cases. For retinopathy, there is no such apparent linkage.

Thus, we assume that smoking, obesity and glycemic control are risk factors with a very variable impact on the actual—microvascular—pathomechanistic aspects of periodontitis, while confirmation of nephropathy and/or neuropathy indicates a definite onset of microangiopathy. We also saw an additive effect of multiple complications on assumed periodontitis, which was previously reported for periodontitis severity [45]. DM patients, who appear to be unaffected by nephropathy and retinopathy, also have a low risk for periodontitis [52]. Due to the cross-sectional nature of our assessment, it is also possible that periodontitis contributes to the onset of diabetic microvascular complications rather the other way round [53]. In PARADIES, we observed representative overall frequencies of microvascular complications; thus, larger statistical power may be possibly needed in order to isolate all these complications as independent predictors.

Even though the strongest predictor for DM complications is DM duration, our adjusted model for clinically diagnosed periodontitis only fairly supports the assumption that long-term metabolic impairment contributes to the crucial systemic vascular and immunological damage. Previous studies reported that both microvascular complications and DM duration are periodontitis risk factors for prevalence and severity [38, 45, 54–56]. Age, smoking, obesity and antihyperglycemic therapy may be weaker surrogates for microvascular complications.

Oral hygiene and periodontitis are clearly connected. A meta-analysis on abundant and consistent studies shows this detrimental effect of poor oral hygiene and avoided dentist appointments on periodontitis [57], and was confirmed recently [58, 59]. At least for assumed periodontitis, our data support this notion.

Similarly, quality of life, especially in relation to oral health, does not always seem to be negatively associated with periodontitis [60, 61]. Still, many papers find a connection between gingival and parodontal disease with impaired general well-being, especially in, but not limited to patients with T2DM [62–65]. The PARADIES study found inconsistent associations between oral health perception (MLQ) and diabetes-related quality of life with clinically diagnosed, respective assumed periodontitis. Impaired quality of life in specific relation to oral health (MLQ) seems to be the stronger component when assessing periodontitis based on the CPI.

Our study provides several strengths. Our DM cohort is of considerable size, with only few studies of comparable statistical power in the previous literature (*n* = 75; *n* = 327; *n* = 300) [66–68]. Other than in most previous studies, PARADIES subjects were recruited in their diabetological environment and not in the context of dental treatment. Thus, we are able to evaluate patients, who refrain from regular dentist appointments. We also managed to achieve a participation rate of 78% without considerable differences between participants and declining patients, assuring generalizable data. Women, men and both patients with T1DM and T2DM are well represented. CPI measurements in the study location (*n* = 150) were taken by the same examiner and in most cases matched the results of the external dentist, showing low observer-dependent variability of objective periodontitis diagnosis. We used validated questionnaires (PAID, MLQ) and successfully re-validated the modified AAP risk score by demonstrating overlapping results for both clinically diagnosed and assumed periodontitis.

There are also limitations to our project. Given the cross-sectional nature of PARADIES, we are unable to assess causality for reported associations. In particular, linkage to microvascular complications may be bidirectional. However, our analysis provides plausible data which warrant further investigation. We did not collect data on educational or social status, limiting the possibility to extrapolate risk profiles to socioeconomic parameters. The recently developed German Periodontitis Screening Questionnaire includes factors such as educational level, which would have been an additional parameter for our analysis. However, the questionnaire was not published, when the PARADIES trial was started [69]. Future studies should include this assessment in order to distinguish the effects of diabetogenic lifestyle, access to medical care and medical knowledge, as well as mere metabolic factors. We also point out that only 39% of our patients provided objective CPI data on their periodontitis status. CPI measurement itself can be biased, as the loss of dental attachment is not a criterion and only partial mouth recording protocol applies to this method [70]. As another disadvantage CPI measures only one tooth per oral sextant, which might lead to both over- and underestimation of periodontitis frequency. Other periodontitis assessments would include the precise depth of gum pockets, loss of attachment and even X-ray results, which are not feasible for screening in a non-dentist environment. However, the specifically designed WHO CPI probe, provides an easy and quick opportunity to assess periodontitis state in typical screening conditions. We also assumed that CPI would be available from our patients’ dentists, allowing an internal validation by comparison of the measurements, done within the course of the study and assessments, which were provided by the patients’ original dentist. They also used modified AAP risk score had a low-to-moderate overlap with the CPI result, leaving some uncertainty of the diagnosis for both tools. Still, results on risk indicators for both assessments did not point into different directions. We assessed DMFT values without radiological examinations, which might underestimate invisible dental destructions. Nevertheless, DMFT did not play a major role in our study, as there are lots of other factors contributing to loss of teeth.

In summary, we replicate that age, smoking, male sex, poor glycemic control and poor orodental care are numerical contributors to the presence of periodontitis. We extend this knowledge by showing that oral health-related quality of life and maybe long-term complications seem to be more specific predictors of periodontitis in DM patients. Microvascular complications are not a diabetes-specific problem, but may occur in patients of older age, with smoking history or chronic poor oral hygiene. Thus, a replication analysis of our assessment in a non-diabetic cohort is warranted.

## Supplementary Information

Below is the link to the electronic supplementary material.

Supplementary file1 (DOCX 30 KB)

## Data Availability

Data sets are available by request to the corresponding author.

## Abbreviations

AAP: American Association of Periodontology
AGE: Advanced glycemic endproducts
CPI: Community Parodontal Index
DM: Diabetes mellitus
DMFT: Index for decayed, missing or filled teeth
DMS-V: Deutsche mundgesundheits-studie V (German oral health study)
MLQ: Mundgesundheits-bezogene lebens-qualität (oral-related quality of life)
OHIP: Oral health impact profile
PAID: Problem areas in diabetes
PARADIES: Periodontitis, age-related DISEASES and diabetes in an endocrinological outpatient setting
SIDD: Severe insulin-deficient diabetes
T1DM: Type 1 diabetes mellitus
T2DM: Type 2 diabetes mellitus
