# Supplementary material for: Periodontitis, age-related diseases and diabetes in an endocrinological outpatient setting (PARADIES): a cross-sectional analysis on predictive factors for periodontitis in a German outpatient facility
Source: Acta Diabetol. 2022 Jan 4;59(5):675–86. doi: 10.1007/s00592-021-01838-z (PMC8995287; doi:10.1007/s00592-021-01838-z)
Supplement: Supplementary file 1 — Supplementary file1 (DOCX 30 KB) [file 592_2021_1838_MOESM1_ESM.docx]

Supplemental Figure 1: Prediction of periodontitis by the modified AAP risk score


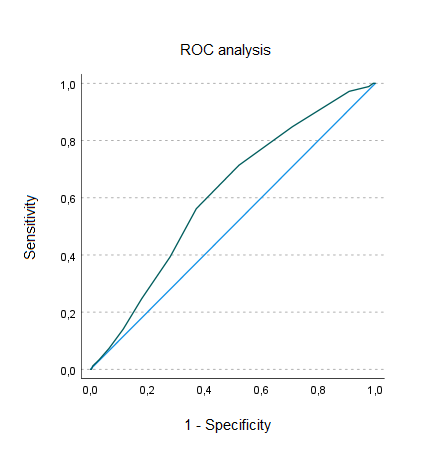


AAP: risk score by the American Association of Periodontology; CPI: Community Parodontal Index; Clinically diagnosed periodontitis: CPI>2; ROC analysis for the prediction of clinically diagnosed periodontitis (CPI>2) by the modified AAP risk score. AUROC 0.610; sensitivity 71 %, specificity 48 %, p<0.001

Supplemental table 1: Association between HbA1c and periodontitis severity

|  | T1DM  HbA1c < median | T1DM  HbA1c > median | T2DM  HbA1c < median | T2DM  HbA1c > median |
| --- | --- | --- | --- | --- |
| Patients with CPI (n=461) | | | | |
| CPI=1 | 0/40 (0.0 %) | 0/40 (0.0 %) | 0/190 (0.0 %) | 0/190 (0.0 %) |
| CPI=2 | 16/40 (40.0 %) | 14/40 (35.0 %) | 78/190 (41.1 %) | 75/191 (39.3 %) |
| CPI=3 | 18/40 (45.0 %) | 19/40 (47.5 %) | 73/190 (38.4 %) | 71/191 (37.2 %) |
| CPI=4 | 6/40 (15.0 %) | 7/40 (17.5 %) | 39/190 (20.5 %) | 45/191 (23.6 %) |
| Patients with AAP risk score (n=1180) | | | | |
| Risk score ≤ 21 | 32/85 (37.6 %) | 31/84 (36.9 %) | 181/506 (35.8 %) | 150/505 (29.7 %) |
| Risk score >21 | 47/85 (55.3 %) | 44/84 (52.4 %) | 285/506 (56.3 %) | 319/505 (63.2 %) |
| Risk score >26 | 6/85 (7.1 %) | 9/84 (10.7 %) | 40/506 (7.9 %) | 36/505 (7.1 %) |

AAP: risk score by the American Association of Periodontology; CPI: Community Parodontal Index; Clinically diagnosed periodontitis: CPI>2; assumed periodontitis: modified AAP risk score > 21 points; Two-sided X²-tests comparing patients above and below the median HbA1c. No significant differences were found.

Supplemental table 2: Association between smoking status and periodontitis severity

|  | T1DM  smokers | T1DM  non-smokers | T2DM  smokers | T2DM  non-smokers |
| --- | --- | --- | --- | --- |
| Patients with CPI (n=449) | | | | |
| CPI=1 | 0 (0.0 %) | 0 (0.0 %) | 0 (0.0 %) | 0 (0.0 %) |
| CPI=2 | 8/18 (44.4 %) | 23/58 (39.7 %) | 19/55 (34.5 %) | 130/318 (40.9 %) |
| CPI=3 | 7/18 (38.9 %) | 26/58 (44.8 %) | 18/55 (32.7 %) | 124/318 (39.0 %) |
| CPI=4 | 3/18 (16.7 %) | 9/58 (15.5%) | 18/55 (32.7 %) | 64/318 (20.1 %) |
| Patients with AAP risk score (n=1180) | | | | |
| Risk score ≤ 21 | 8/49 (16.3 %) | 55/120 (43.7 %) *** | 12/166 (7.2 %) | 319/845 (37.8 %) *** |
| Risk score >21 | 30/49 (61.2 %) | 61/120 (48.4 %) *** | 118/166 (71.1 %) | 486/845 (57.5 %) *** |
| Risk score >26 | 11/49 (22.4 %) | 4/120 (3.2 %) *** | 36/166 (21.7 %) | 40/845 (4.7 %) *** |

AAP: risk score by the American Association of Periodontology; CPI: Community Parodontal Index; Clinically diagnosed periodontitis: CPI>2; assumed periodontitis: modified AAP risk score > 21 points; Two-sided X²-tests comparing smokers vs. non-smokers are significant with * p<0.05; ** p<0.01; *** p<0.001.
